# Immunomodulatory Proteins in Tick Saliva From a Structural Perspective

**DOI:** 10.3389/fcimb.2021.769574

**Published:** 2021-10-13

**Authors:** Stepan S. Denisov, Ingrid Dijkgraaf

**Affiliations:** Department of Biochemistry, Cardiovascular Research Institute Maastricht (CARIM), University of Maastricht, Maastricht, Netherlands

**Keywords:** protein, saliva, immunomodulatory, ticks, structure-activity relationship

## Abstract

To feed successfully, ticks must bypass or suppress the host’s defense mechanisms, particularly the immune system. To accomplish this, ticks secrete specialized immunomodulatory proteins into their saliva, just like many other blood-sucking parasites. However, the strategy of ticks is rather unique compared to their counterparts. Ticks’ tendency for gene duplication has led to a diverse arsenal of dozens of closely related proteins from several classes to modulate the immune system’s response. Among these are chemokine-binding proteins, complement pathways inhibitors, ion channels modulators, and numerous poorly characterized proteins whose functions are yet to be uncovered. Studying tick immunomodulatory proteins would not only help to elucidate tick-host relationships but would also provide a rich pool of potential candidates for the development of immunomodulatory intervention drugs and potentially new vaccines. In the present review, we will attempt to summarize novel findings on the salivary immunomodulatory proteins of ticks, focusing on biomolecular targets, structure-activity relationships, and the perspective of their development into therapeutics.

## Introduction

In 2020, a monument to ticks has been erected in the Russian city of Ufa. The tick-casted from silver-sits on a top of a half meter high stone from the Ural Mountains with the inscription: “Same as you I also want to live”, which according to the artists should underline that ticks are a part of nature despite negative connotations. Contrary to the inscription that translates some feeling of endangerment, ticks seem to be thriving. They count over 900 species in three families: *Ixodidae* (hard ticks), *Argasidae* (soft ticks) and monospecific *Nuttalliellidae* (Gugliemone et al., 2010; Dantas-Torres, 2018). Tick habitat is expanding due to climate change accelerating development and shortening tick life cycles due to increasing temperature and earlier springs (Ogden and Lindsay, 2016). The intensification of the global transport of goods, humans, and animals leads to the introduction of invasive tick species worldwide (Makenov et al., 2021; Molaei et al., 2021; Tufts and Diuk-Wasser, 2021). In what the Russian artists from Ufa are without a doubt right, that ticks as an indispensable part of nature are our neighbors and encounters with them will become more frequent in the future. In that light, it is of the utmost importance to study ticks and their adaptation mechanisms in order to combat, control and in the best case even use them to our advantage.

Ticks are blood-sucking parasites which require a notoriously long time to acquire a blood meal that can take up to two weeks. During all this time, ticks must counteract the host’s defense mechanisms, ranging from blood coagulation and immune responses to grooming. The main evolutionary adaptation for this is a rich collection of bioactive compounds in the saliva of ticks, many of which are of a polypeptide nature with diverse activity, such as anticoagulant, immunomodulatory, vasodilatory, etc. (Kazimírová and Štibrániová, 2013). These proteins could be used in the development of anti-tick vaccines and novel bioinspired therapeutics (Aounallah et al., 2020; van Oosterwijk and Wikel, 2021). In this review, we will provide an overview of tick salivary proteins with immunomodulatory activity focusing on their structure-activity relationships.

## Evasins

The chemokine signaling system is an integral part of homeostasis and inflammatory responses as it regulates cell trafficking and recruitment. In humans, this system includes 46 small secreted proteins – chemokines (chemotactic cytokine) – and 23 chemokine receptors (Bachelerie et al., 2013). Pathogens and parasites in particular viruses, worms, and ticks target the chemokine signaling to suppress the host immune response (Proudfoot et al., 2015). Ticks, in particularly, use evasins – a unique class of cysteine-rich chemokine-binding proteins (Déruaz et al., 2008). Recently, they have been comprehensively reviewed by Bhusal et al., (2020) to which we refer interested readers for in-depth information. Here, we give a brief summary and discuss novel research articles that have been published since then.

**Figure 1:**
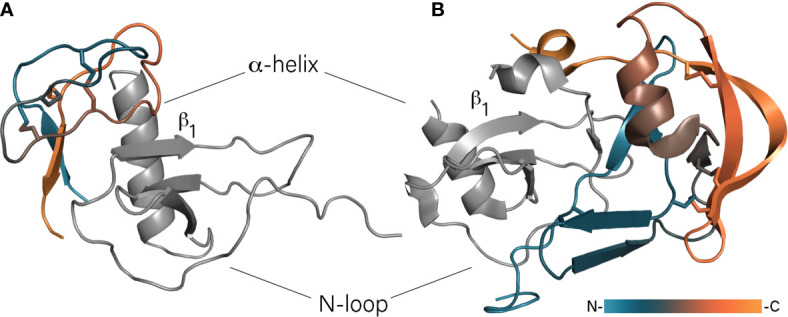
Binding of chemokines by class A and B evasins. **(A)** The model of EVA-3/CXCL8 complex. Unstructured *N*- and *C*-termini of EVA-3 are hidden for visibility. **(B)** The crystal structure of EVA-1/CCL3 complex (PDB: 3FPU). Evasins are colored according to a gradient from *N*- to *C*-terminus, chemokines are shown in grey and oriented in the same direction.

The evasin family, initially discovered in *Rhipicephalus sanguineus*, currently numbers several hundred putative members from soft and hard tick genera (Hayward et al., 2017; Singh et al., 2017). Evasins fall into two structurally unrelated groups – class A and B, which preferentially bind CC- and CXC-type chemokines, respectively (Bhusal et al., 2020). Class A evasins are proteins of 89 – 126 amino acid residues and usually contain eight cysteines that form four disulfide bonds. Obtained crystal structures of EVA-1 (also known as evasin-1), its complex with CCL3 (**Figure 1B**), and EVA-4 (evasin-4) indicate that the class A evasins are specific for ticks and have no structural analogs in mammals (Dias et al., 2009; Denisov et al., 2020b). The structures of both class A proteins – EVA-1 and EVA-4 – consist of seven or eight β-strands and one short α-helix forming *N*- and *C*-terminal subdomains. These subdomains are arranged in a boat-shaped structure accommodating a monomeric chemokine ligand. The interaction of EVA-4 with chemokines is mainly facilitated by the flexible *N*-terminus that binds in the groove between the *N*-loop and β_3_-strand of the chemokine (Denisov et al., 2020b). EVA-1 binding to chemokines includes not only contacts of the *N*-terminus, but also interaction of its *C*-terminus with the *N*-terminus of the particular CC-type chemokine (Dias et al., 2009). This additional interaction could explain the higher selectivity of EVA-1 which binds CCL3, CCL4, and CCL18, in contrast to EVA-4 which binds more than 20 CC-type chemokines (Frauenschuh et al., 2007; Déruaz et al., 2013).

The class B evasins are 60 – 100 amino acid residue proteins with six very conservative cysteines and, unlike class A evasins, bind chemokines of the CXC-type. The first discovered and best studied class B evasin – EVA-3 (evasin-3) – binds CXCL1, -2, -3, -5, -6, and -8 (Déruaz et al., 2008; Lee et al., 2019). The structure of EVA-3 has been solved by both X-ray crystallography and NMR spectroscopy and has revealed the presence of a rigid structured core and flexible *N*- and *C*-termini (Denisov et al., 2019a; Lee et al., 2019). The unstructured termini contain multiple *N*- and *O*-glycosylation sites that do not participate in chemokine binding as their truncation affects neither binding nor inhibitory activity (Denisov et al., 2019a). The core of the protein consists of an almost orthogonally oriented short two-stranded antiparallel β-sheet and two loops stapled together by three disulfide bonds. These disulfide bonds are arranged in the so-called inhibitory cysteine knot (ICK) in which one disulfide bond protrudes through the cycle formed by two others (Denisov et al., 2019a; Denisov et al., 2019b; Lee et al., 2019). With CXCL8, EVA-3 forms a 1:1 complex in which the β-sheet of EVA-3 intercalates between the CXCL8’s α-helix and *N*-loop and the bigger EVA-3 loop interacts with the β_1_-strand of CXCL8 (**Figure 1A**) (Denisov et al., 2019a). In that way, EVA-3 disrupts the CXCL8 dimer and causes intramolecular rearrangement of CXCL8, preventing interaction with chemokine receptors. In addition, the intercalation of EVA-3 between the helix and *N*-loop of CXCL8 disrupts its glycosaminoglycan (GAG)-binding site, possibly causing dissociation of cell wall bound CXCL8. In contrast to CXCL8, CXCL1 has two GAG-binding sites and in case of binding of this chemokine by EVA-3, one site remains available for GAG-binding. In that case, EVA-3 binds directly to GAG-bound CXCL1 deposited on cell walls (Denisov et al., 2020a).

Although the chemokine signaling system is involved in a broad variety of physiological and pathophysiological processes, targeting it as a therapeutic approach is challenging due to a high level of redundancy within the signaling system where one receptor is activated by multiple chemokines and vice versa (Horuk, 2009). Despite the existence of two dozen chemokine receptors, only three drugs are currently on the market that target these receptors: Maraviroc, Plerixafor, and Mogamulizumab (Hughes and Nibbs, 2018), what underscores the difficulties in blocking chemokine signaling by receptor antagonists. In that light, targeting chemokines instead of chemokine receptors using chemokine-binding agents including evasins could be an alternative strategy (Proudfoot et al., 2015; Bhattacharya and Kawamura, 2020).

EVA-3 has been found to decrease inflammation in a mouse model of acute pancreatitis and myocardial infarction (Montecucco et al., 2010; Montecucco et al., 2014). The ICK motif embodied in the EVA-3 structure is a prospective scaffold for drug development due to its exceptional proteolytic stability and high tolerance to sequence variations and chemical modifications (Craik et al., 2001). Taking into account that two ICK-containing peptides – Ziconotide (Jain, 2000) and Linaclotide (Rothstein and Friedenberg, 2013) – are currently on the market, class B evasins can be attractive candidates for further drug development. Although EVA-1 and EVA-4 have effectively diminished inflammation in the murine model of acute lung injury and myocardial infarction, respectively (Russo et al., 2011; Braunersreuther et al., 2013), their application as drugs could be challenging due to the complex structures and difficult production (personal observation). However, it has recently been shown that short linear and cyclic peptides derived from the *N*-termini of EVA-P672 and EVA-4 retain binding and inhibitory activity comparable to full-length parent proteins (Darlot et al., 2020; Denisov et al., 2020b). Moreover, these peptides could be further modified and adjusted for neutralization of chemokines of interest. To provide an example, derived peptides contain Tyr residues, which could be sulfated. This modification has been shown to increase the affinity of the homological evasin ACA-01 from *Amblyomma cajennense* to particular chemokines presumably mimicking the interaction with chemokine receptors (Franck et al., 2020).

## Serpins

Serpins, serine protease inhibitors, are a superfamily of 40 – 50 kDa proteins found in mammalians, insects, plants, fungi, prokaryotes, and viruses (Lucas et al., 2018). Being recognized as a protein family in 1980 (Hunt and Dayhoff, 1980), over 1500 members are known to date. Abundant structural data of serpins, including almost 200 X-ray crystal structures from more than 30 different organisms (Mahon and McKenna, 2018), provide detailed insight into the structure-activity relationships of serpins. Despite low sequence homology, all serpins adopt a similar three-dimensional fold embodying the *N*-terminal helical and *C*-terminal β-barrel domains (Huntington, 2011). The latter consist of three β-sheets named A – C and the long flexible reactive center loop (RCL) containing the scissile bond between residues P1 and P1’. Upon cleavage of this bond, the RCL undergoes a conformational change and incorporates itself into β-sheet A, forming a hyperstable relaxed conformation. As a result, a serine protease, remaining covalently attached to a P1 residue in the form of an acyl-enzyme complex, is moved away from the top of a serpin. That causes distortion of a protease’s catalytic triad and its inability to hydrolyze an ester bond between serine and P1 resides, making binding irreversible (suicidal). Serpins whose RCL cannot form a hyperstable insert to the β-sheet, lack classical inhibitory activity, but act as transporters and regulators of blood pressure and vasomotor activity (Lucas et al., 2018).

Due to the suicidal nature of serpin binding, serpins play a crucial role in enzymatic cascades where tight regulation is necessary such as in blood coagulation and activation of the complement system. Blood-sucking arthropods, including ticks, acquired diverse serpins in their saliva to counteract the host hemostasis and immune responses (Meekins et al., 2017) with most of the discovered tick serpins described as having anti-hemostatic functions. As this activity is beyond the scope of the present review, we refer interested readers to dedicated reviews (Blisnick et al., 2017; Chmelař et al., 2017) and focus on serpins with prominent immunomodulatory activity.

**Figure 2:**
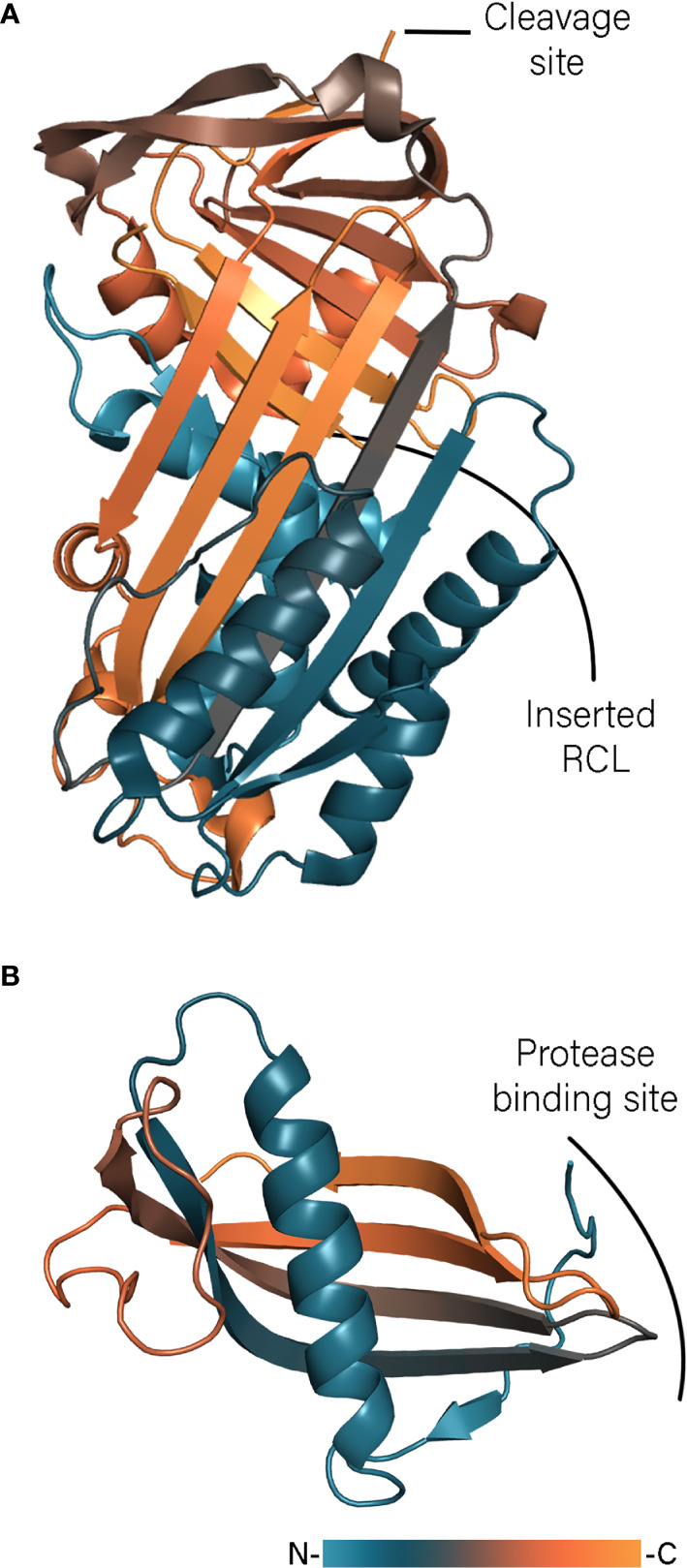
Tick serpin Iripin-3 (**A**, PDB: 7AHP) and cystatin Sialostatin L2 (**B**, PDB: 3LH4) structures colored according to a gradient from *N*- to *C*-terminus.

The best characterized immunomodulatory serpins are isolated from *Ixodes ricinus*. *Ixodes ricinus* immunosuppressor – Iris – has been shown to suppress T lymphocyte production and inhibit expression of TNF-α, IFN-γ, and IL-6 (Leboulle et al., 2002). Iris possesses an α1-antitrypsin-like activity inhibiting human leukocyte elastase (HLE), tissue plasminogen activator (t-PA), thrombin and factor Xa (FXa) (Prevot et al., 2006). The inhibition profile depends on the sequence of the RCL and could be changed to anti-thrombin-like inhibition by mutation of a Met to an Arg residue in the P1 position. Interestingly, the immunomodulatory activity of Iris cannot simply be attributed to inhibition of pro-inflammatory enzymes such as HLE. It has been shown that the inactive serpin variant L339A, with a mutated P2 residue, inhibited the release of inflammatory cytokines as effective as the native protein (Prevot et al., 2009). This independent enzyme inhibition activity is most likely facilitated through direct interaction of Iris D and E helices with monocytes. Immunization studies with Iris in rabbits resulted in 30% mortality and reduced weight gain of both adult ticks and nymphs, indicating a low level of protection and making Iris a poor vaccine candidate (Prevot et al., 2007). In contrast to Iris, *Ixodes ricinus* serpin-2 (IRS-2) embodies Tyr as the P1 residue and therefore possesses anti-chymotrypsin activity (Chmelar et al., 2011). IRS-2 inhibits cathepsin G and chymase produced by neutrophils and mast cells, respectively and blocks the IL-6/STAT-3 signaling pathway (Chmelar et al., 2011; Páleníková et al., 2015). The latest addition to serpins from *Ixodes ricinus* are Iripin-3, -5, and -8 (Chlastáková et al., 2021; Kascakova et al., 2021; Kotál et al., 2021) Iripin-3 reduces IL-6 production and inhibits the Th1 immune response (Chlastáková et al., 2021) and Iripin-5 inhibits macrophages migration and blocks activation of the complement (Kascakova et al., 2021). Although both serpins contain an Arg residue in the P1 position and show anti-trypsin activity, Iripin-3 blocks kallikrein and matriptase whereas Iripin-5 inhibits human neutrophil elastase and proteinase 3. The crystal structures of IRS-2, Iripin-5, and Iripin-3 (**Figure 2A**), obtained in the relaxed variant of which the cleaved RCL is inserted into the β-sheet, revealed classical serpin fold with three β-sheets and 10 and 9 α-helices, respectively (Chmelar et al., 2011; Chlastáková et al., 2021; Kascakova et al., 2021). In contrast the crystals of Iripin-8 is obtained in its native form shedding light on the structure of the RCL region (Kotál et al., 2021). The RCL of Ipirin-8 is unusually long comparing with other Iripins and contains a type II polyproline helix, which brings the P1 residue away from the protein core. That is hypothesized that such protruded RCL could facilitate binding of some unknown tick protease and therefore regulates physiological processes of tick itself.

Several other immunomodulatory serpins from other hard ticks have been identified and studied as well. The homolog of Iris *Ixodes persulcatus* immunosuppressor 1 (Ipis-1) diminishes production of IFN-γ by direct interaction with T cells (Toyomane et al., 2016). Multiple serpins have been identified in *Amblyomma americanum* (Mulenga et al., 2007), two of which – AAS27 and AAS41 – have been shown to suppress inflammatory responses (Tirloni et al., 2019; Kim et al., 2020). AAS41 inhibits chymase and chymotrypsin, whereas AAS27 blocks trypsin and trypsin-like serine protease such as chymotrypsin, factor XIa, and plasmin. Both proteins effectively reduced chymase-mediated inflammation induced by 48/80 (an agonist of mast cell degranulation) in rats. Interestingly, althoughAAS46 and AAS41 have 97% identity, AAS41 has moderate to no inhibitory activity against the tested panel of enzymes, indicating a different function for this serpin (Kim et al., 2020). Serpins from *Haemaphysalis longicornis* named HlSerpin-a and HlSerpin-b inhibit the activity of cathepsin G and factor Xa and abrogate LPS-induced mRNA transcription of TNF-α, IL-6, and IL-1β in mouse bone-marrow-derived dendritic cells and macrophages (Wang et al., 2020). Remarkably, a 20 amino acid peptide derived from the RCL of HlSerpin-a could impair cytokine production and diminished inflammation in a mouse arthritis model. Several serpins from *Rhipicephalus microplus* (RmS) have been shown to decrease production of IFN-γ and metabolic activity of Con A-stimulated spleen cells (Coutinho et al., 2020).

## Cystatins

Cystatins are another superfamily of protease inhibitors, which, unlike serpins, reversibly inhibit cysteine proteases. Initially discovered in chicken egg white, cystatins occur almost ubiquitously in birds, insects, plants, mammalians, and humans (Turk et al., 2008). Human cystatins inhibit cysteine proteases called cathepsins and are involved in multiple (patho)physiological processes including tumor progression, apoptosis, cerebrovascular amyloid deposition, antigen presentation, NO and cytokine release. (Shamsi and Bano, 2017). Cystatins share a common cystatin fold composed of a five-stranded antiparallel β-sheet wrapped around a *N*-terminal α-helix (Turk et al., 2008). Based on sequence similarity and the presence of specific cystatin-like segments in human cystatins, three protein clades could be distinguished (Abrahamson et al., 2003): (I) type I cystatins – also known as stefins – which are intracellular proteins consisting of ~100 amino acid residues without disulfide bonds or glycosylation; (II) type II cystatins that consist of ~120 amino acid residues containing two conservative disulfide bridges; and (III) type III cystatins or so-called kininogens which are high molecular weight proteins due to presence of three type II cystatin-like domains and glycosylated side chains. The binding to proteases is determined by three cystatin regions, namely: the conservative Gly residue in the *N*-terminus and the Gln-Xaa-Val-Xaa-Gly and Pro-Trp motifs of the β-hairpin loop 1 and 2, respectively – which together form a wedge-shaped edge (Abrahamson et al., 2003).

In ticks, cystatins are mainly expressed in the midgut or saliva where they regulate proteolytic activity of ingested host factors and immune responses (Schwarz et al., 2012). The best-studied tick salivary cystatins are two close homologs proteins from *Ixodes scapularis*, sialostatins L and L2. Although the name of sialostatin L is due to its ability to inhibit cathepsin L (Kotsyfakis et al., 2006), it also inhibits cathepsin L, S, V, and C, while sialostatin L2 only shows higher inhibitory activity towards cathepsin L and V (Kotsyfakis et al., 2007). The crystal structure of sialostatin L2 revealed that the protein adopts a common type II cystatin fold (**Figure 2B**) (Kotsyfakis et al., 2010). In contrast to a monomeric form of sialostatin L2, L crystallized in a similar way to human cystatin C, namely as a dimer formed by domain swapping (Shamsi and Bano, 2017). Although cystatin C dimerization has been implicated in development of hereditary cystatin C amyloid angiopathy (Turk et al., 2008), in the case of sialostatin L only traces of a dimeric form have been observed even at high concentrations, indicating that only the monomeric form is physiologically relevant (Kotsyfakis et al., 2010). Modeling of the monomer of sialostatin L showed the same fold as for L2. However, the *N*-terminal region of L2 adopt different conformations and is packed against the β-sheet instead of forming an extended coil or loop, which could explain the different inhibitory specificity. Sialostatins have been shown to modulate the activity of dendric cells in different ways. Sialostatin L inhibits LPS-induced maturation of dendric cells (Klein et al., 2015), whereas L2 reduces the expression of inflammatory chemokines (Lieskovská et al., 2015a; Lieskovská et al., 2015b). Sialostatin L reduces the expression of IL-9 through IL-1R1 signaling alleviating asthma symptoms (Horka et al., 2012; Klein et al., 2015). Sialostatin L2 inhibits inflammasome formation indirectly by targeting caspase-1 activity (Chen et al., 2014). This activity is independent of protease inhibition and is mediated by binding of the loop 2 of sialostatin L2 with annexin A2 (Wang et al., 2016a).

Several other salivary cystatins from hard ticks have identified and characterized as well. Two sialostatin orthologs from *Ixodes persulcatus* – Ip-sL1 and Ip-sL2 – have been found to inhibit cathepsins L and S and lower the expression of inflammatory agents by dendric cells (Sajiki et al., 2020). In the same tick species, a putative immunomodulatory cystatin JpIpcys2b has been identified and proposed to inhibit cathepsin L (Rangel et al., 2017). Iristatin isolated from *Ixodes ricinus* is a potent inhibitor of cathepsin L and C, which attenuates both the Th1 and Th2 immune response (Kotál et al., 2019). The crystal structure of Iristatin showed that the β-sheet of the classical cystatin fold is distorted in Iristatin and contains only four β-strands lacking the *N*-terminal one. HlSC-1 isolated from *Haemaphysalis longicornis* inhibits cathepsin L (Yamaji et al., 2009), whereas BrBmcys2b from *Rhipicephalus micropolus* showed broad inhibitory activity blocking cathepsins L, B, and C (Parizi et al., 2015). The mechanism of immunomodulatory activity has been deciphered for DsCystatin from *Dermacentor silvarum* (Sun et al., 2018). It has been shown that DsCystatin directly targets cathepsin L and B, attenuating TLR4 signaling and impairing expression of TRAF6.

Cystatins from soft ticks are less studied and only two members from *Ornithodoros moubata* are described – OmC1 and OmC2 mainly expressed in gut and salivary glands, respectively (Grunclová et al., 2006). In contrast to sialostatin L, OmC2 inhibits not only cathepsins L, S, and C, but also blocks lysosomal cathepsins B and H (Salát et al., 2010). The high level of flexibility of the *N*-terminus observed in the resulting crystal structure could contribute to the broad selectivity of OmC2. OmC2 suppresses antigen presentation and TNF-α and IL-12 production through inhibition of cathepsins L and S (Salát et al., 2010; Zavašnik-Bergant et al., 2017). Tick cystatins have been probed as antigens in vaccination studies. Immunization against OmC2 in guinea pigs increased mortality of nymphs after engorgement (Salát et al., 2010). On the contrary, immunization against sialostatin L2 increased the rejection rate and delayed ticks’ drop-off and impaired overall feeding success (Kotsyfakis et al., 2008).

## Lipocalins

Lipocalins comprise the vast and diverse family of proteins that carry lipophilic molecules and are found in vertebrates, insects, plants, and bacteria (Grzyb et al., 2006). Being part of a larger calycin superfamily, their distinguishing feature is the formation of the hydrophobic cup-shaped β-barrel to accommodate a lipophilic molecule, which is reflected in the name derived from Greek “Λιποζ” – fat and “καΛυξ” – a calyx, pod. The calyx is formed by eight β-strands (labeled A – H) connected by seven loops (L1 – L7). Strand A is preceded by a short 3_10_-helix, which is characterized by a tight turn containing 3 amino acid residues in contrast to 3.6 residues in a regular α-helix. Taking together with a longer α-helix following strand H, they form the classical lipocalin fold (Flower et al., 1993). Due to a high level of structural and sequence variations among lipocalins, three structural conservative regions (SCRs) within the fold are designated, namely 3_10_-helix and strand A (SCR1), strands F, G, and connecting loop L6 (SCR2), and strand H and the part of following loop (SCR3). Lipocalins fall into two subfamilies: kernel lipocalins where all three SCRs are present, and outlier lipocalins where only part of them are conserved (Flower, 1996).

Lipocalins are widely employed by blood-sucking arthropods, including soft and hard ticks, as antihemostatic agents with wide variety of activities such as reducing inflammation, preventing blood coagulation and platelet aggregation (Montfort et al., 2000). In soft ticks, lipocalins were initially identified as 15 – 17 kDa components of salivary gland granules in *Ornithodoros savignyi* and named tick salivary gland proteins (TSGPs) (Mans et al., 2001; Mans et al., 2002). Although structure modeling of TSGPs showed a lipocalin fold with an eight-stranded antiparallel β-barrel and two α-helices, none of these proteins have SCRs and therefore are classified as members of the outlier subfamily (Mans et al., 2003). TSGPs and other soft tick lipocalins fall into several groups according their function and sequence: serotonin and histamine scavengers (TSGP1); and cysteinyl leukotriene scavengers (TSGP4); complement inhibitors, thromboxane A2 (TXA_2_) and leukotriene B_4_ (LTB_4_) scavengers (TSGP2/3) (Roversi et al., 2007; Mans et al., 2008; Mans and Ribeiro, 2008a). Lipocalins from hard ticks have been found to sequester histamine, LTB_4_, and cholesterol (Paesen et al., 1999; Beaufays et al., 2008a; Roversi et al., 2017). Thromboxane A2 scavengers, such as moubatin (Waxman and Connolly, 1993) will be omitted as their function as platelet aggregation inhibitors lies beyond the scope of the current review.

**Figure 3:**
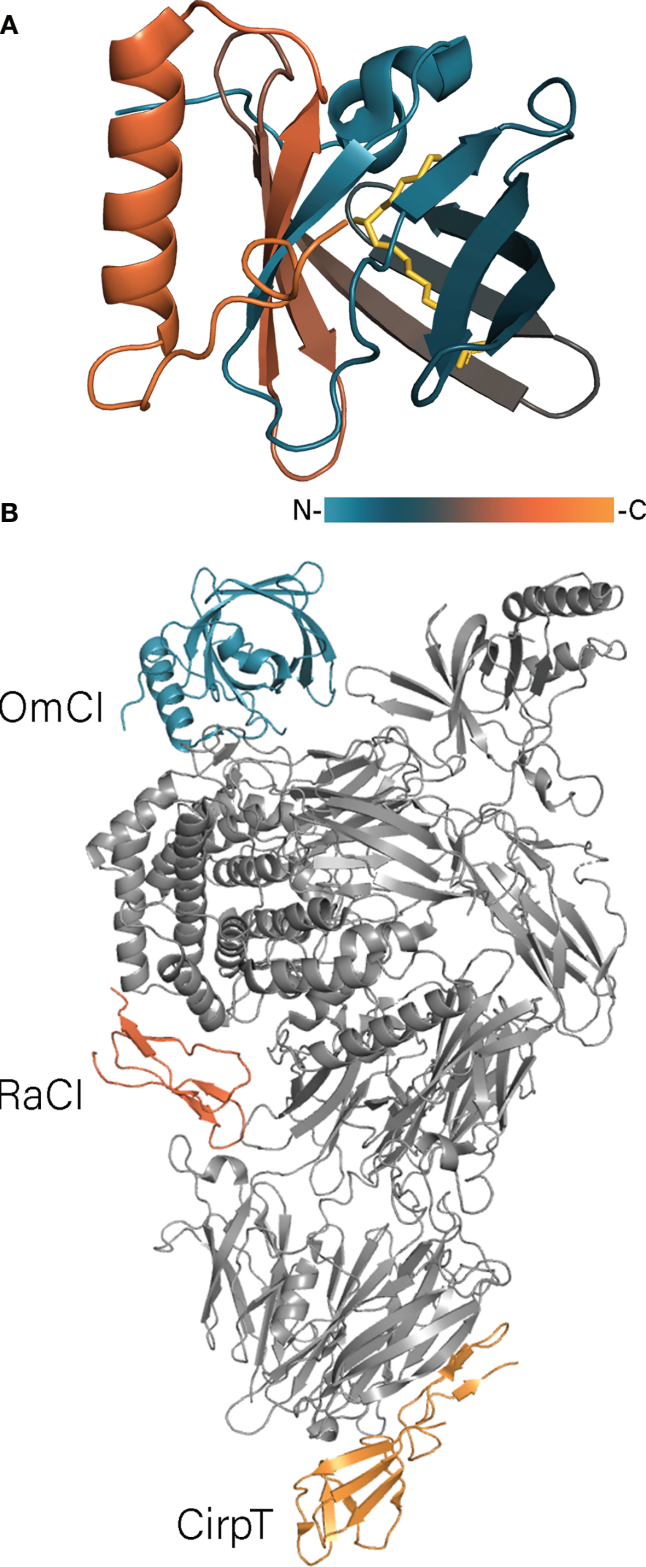
**(A)** OmCI structure in the complex with ricinoleic acid (PDB: 2CM4). OmCI structure is colored according to a gradient, ricinoleic acids is shown in yellow. **(B)** The structure of C5 protein in the complex with OmCi, RaCI, and CirpT (PDB: 6RQJ). C5 is shown in grey, tick proteins – in color.

OmCI (stands for *Ornithodoros moubata* Complement Inhibitory protein) is a 17 kDa lipocalin, which inhibits the complement system of mammals and birds (Nunn et al., 2005; Barratt-Due et al., 2011; Frye et al., 2020). The crystal structure of OmCI (**Figure 3A**) revealed that the protein adopts a lipocalin fold with an eight-stranded antiparallel β-barrel and *N*- and *C*-terminal α-helices (Roversi et al., 2007). The three-dimensional structure is stabilized by three disulfide bonds: C118 – C147 connects the *C*-terminal helix to the calyx, C56-C168 links the *C*-terminal cysteine to the L1 loop, whereas the third C24-C146 bond ties the *N*-terminus to the *C*-terminal α-helix. OmCI has been shown to inhibit complement through high-affinity binding to C5 preventing its cleavage by C5 convertases and thereby the release of C5a (Hepburn et al., 2007; Roversi et al., 2007). In the complex the L3 loop of OmCI makes contacts with the CUB domain of C5 when the H strand together with the C-terminal α-helix of OmCI interact with the C5d domain of C5 (**Figure 3B**) (Jore et al., 2016). Although the C345c domain has been shown to be more closely associated with the C5 core in the complex with OmCI than in the free form (Fredslund et al., 2008), the later crystal structure showed that C345c still remains relatively disordered in the complex and makes one point contact with OmCI (Jore et al., 2016). Besides that, OmCI binds LTB_4_ and ricinoleic acid mostly through the hydrophobic interaction in the calyx (Roversi et al., 2013). The carboxylic group of ricinoleic acid is located in the calyx opening facing solvent, whereas the hydroxylic group is accommodated inside the calyx and contacts the polar Asp and His residues (Roversi et al., 2007). The crystal structures of OmCI-LTB_4_ and OmCI-C5 complexes alongside with SPR data showed that binding of LTB_4_ and C5 are independent and can occur simultaneously (Roversi et al., 2013; Jore et al., 2016). In contrast to TSGP3, neither OmCI nor TSGP2 binds TXA_2_ due to presence of bulky Arg85 in the binding site (Mans and Ribeiro, 2008b). Since LTB_4_ plays a vital role in neutrophil recruitment and migration, its sequestration by lipocalin Ir-LBP from *Ixodes ricinus* has inhibited neutrophil transmigration and delayed their apoptosis (Beaufays et al., 2008b).

OmCI has been shown to be an effective anti-inflammatory agent in multiple inflammation-associated models including sepsis, myocardial infarction, and lung injury (Garcia et al., 2013; Hellerud et al., 2017; Pischke et al., 2017). Being a C5 inhibitor, OmCI has drawn attention as a possible alternative to the already marketed C5 inhibitor eculizumab, which is considered the most expensive drug in the world with an annual treatment price reaching 500k $ per patient. In two case studies, recombinant OmCI (also known as coversin or rEV576, Akari Therapeutics) has been used successfully to treat thrombotic microangiopathy (TMA) associated with hematopoietic stem cell transplantation and paroxysmal nocturnal haemoglobinuria (PNH) in patients with resistance to eculizumab (Goodship et al., 2017; Schols et al., 2020). However, it appeared that in the second case (Schols et al., 2020) an injection of coversin every 12 hours was necessary because of the rapid excretion of the protein from bloodstream. Conjugation of coversin to a long Pro-Ala-Ser polypeptide (PASylation) dramatically increased circulation time without interfering with anticomplement activity (Kuhn et al., 2016). Binding both C5 and LTB_4_ has been shown to have a synergistic inhibitory effect in the murine pemphigoid disease model (Sezin et al., 2019). However, the second-generation drug developed from OmCI – nomacopan – and its variant which binds only LTB_4_ and cannot bind C5 were equally effective in experimental autoimmune uveitis (EAU) (Eskandarpour et al., 2021). Currently, nomacopan is in different phases of clinical trials against bullous pemphigoid, atopic keratoconjunctivis and thrombotic microangiopathies (Nunn and Fettiplace, 2021; Sánchez-Tabernero et al., 2021).

Histamine and serotonin (5-HT) are important mediators of the immune response acting on monocytes, dendritic, and B cells (Jutel et al., 2002; Wu et al., 2019). The presence of histamine- and serotonin-binding lipocalins in both soft and hard ticks indicates the importance of suppressing these mediators, independent of feeding time (Paesen et al., 1999; Mans et al., 2008; Wang et al., 2016b). The crystal structures of monomine (*Argas monolakensis*) and HBP2 (*Rhipicephalus appendiculatus*) with histamine and AM-182 (*Argas monolakensis*) with serotonin are resolved (Paesen et al., 1999; Mans et al., 2008) contributing to our understanding of the ligand binding mode. The crystal structure of monomine and AM-182 showed that the overall folding of both proteins is similar to OmCI (Mans et al., 2008). In contrast to the hydrophobic interior of regular lipocalins, monomine and AM-182 alongside with their hydrophobic contacts employ several charged residues to adopt and form hydrogen bonds with a polar histamine molecule. Unlike monomine and AM-182, which bind one ligand molecule, HBP2 has two binding sites for histamine indicated as L (low affinity) and H (high affinity) (Paesen et al., 1999). The L site coincides with the histamine-binding site of monomine, whereas the H site is located closer to the opening of the calyx. To accommodate histamine in the H site, HBP2 has elongated L1 and L7 loops, which close the opening and form multiple contacts with the ligand (Paesen et al., 2000). TSGP1 has a similar binding mode with two sites, but in contrast to HBP2 which binds two histamine molecules, only the H site binds histamine, while the L site is specific for 5-HT (Mans et al., 2008). The same differentiation of binding site specificity has been also observed for the related HBP2 protein SHBP isolated from *Dermacentor reticulatus* (Sangamnatdej et al., 2002). It is worth to mention that ticks express not only histamine binding lipocalins in saliva, but also tick histamine release factors (tHRF) with the high level of homology to mammalian HRFs (Mulenga et al., 2003; Dai et al., 2010). However, information over tHRFs is very scarce and identification of their exact function requires further investigation.

In addition to LTB_4_, cysteinyl leukotriene E_4_ (LTE_4_) is involved in an inflammatory response that increases monocyte activation. In contrast to LTB_4_, LTE_4_ and its metabolic precursors LTC_4_ and LTD_4_ do not contain hydroxyl groups along a fatty acid alkyl chain. Not surprisingly, the calyx interior of cysteinyl leukotrienes binding TSGP4 is overly hydrophobic (Mans and Ribeiro, 2008a). TSGP4 binds LTC_4_, LTE_4_, and LTD_4_ with nM affinity, whereas the Kd for LTB_4_ binding lies in µM range. Japanin – a member of the novel class of tick lipocalins that bind cholesterol as a ligand – has been described in *Rhipicephalus appendiculatus* (Roversi et al., 2017). Although Japanin and related lipocalins bind dendritic cells and modulate expression of pro-inflammatory agents (Preston et al., 2013), it remains to be investigated whether this activity is due to cholesterol binding.

## Salp15 Family

Salp15 is a 15 kDa protein initially identified in saliva of *I. scapularis* and found to inhibit production of IL-2 and CD4+ T cell activation (Das et al., 2001; Anguita et al., 2002). Dozens of homologs have subsequently been identified in *Ixodes* ticks making up the diverse Salp15 family (Hovius et al., 2007; Mori et al., 2010; Wang et al., 2014; Sultana et al., 2016; Wen et al., 2020). Salp15 and Salp15-like proteins appear to be challenging for structural research as they usually contain seven cysteines making obtaining recombinantly expressed proteins in a single native conformation during oxidative refolding particularly difficult. Using NMR spectroscopy the secondary structure of Salp15 homolog Iric-1 from *Ixodes ricinus* has been partially solved (Kolb et al., 2015). Iric-1 embodies a α-helix in the ^54^Pro-Leu^61^ region and a ^104^Val-Asp^107^ β-strand, whereas the *N*-terminus remains in an unstructured coil-like conformation. Although the C-terminus remains structurally uncharacterized, this region has been shown to interact directly with the CD4 receptor (Garg et al., 2006). Besides immunomodulatory activity, Salp15 draws close attention as it facilitates transmission of *Borrelia*. Salp15 has been shown to bind directly to spirochete’s outer surface protein C (OspC), thereby protecting spirochaetes from antibody-mediated killing (Ramamoorthi et al., 2005; Murase et al., 2015). For in-depth details about the pathogen-tick-host relationships involving Salp15, we would like to refer an interested reader to the recent comprehensive review (Wen et al., 2020).

## TSLPI/Salp14 and Other Complement Inhibitors

Several tick complement inhibitors have been described thus far. However, information about them is often scarce and incomplete to combine them in a particular protein family. Therefore, we will here describe characterized tick salivary complement inhibitors independent of their structural classification.

**Figure 4:**
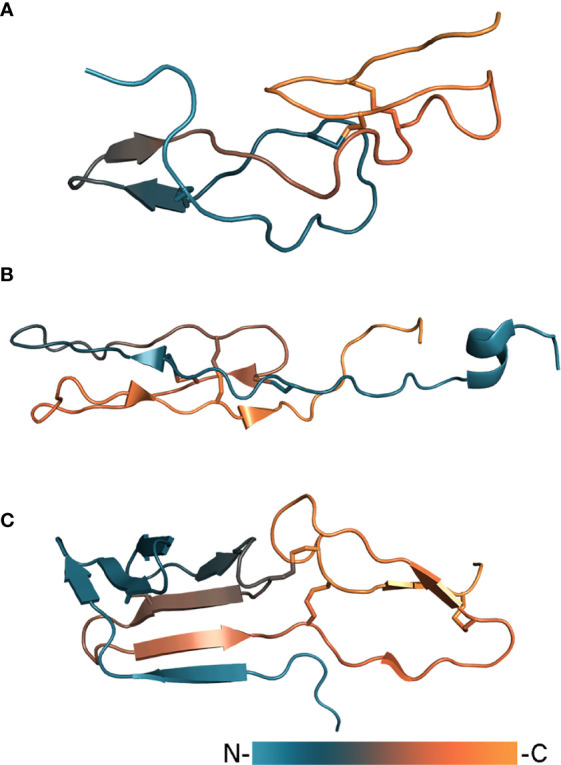
Tick complement inhibitors BSAP1 (**A**, PDB: 7NE8), RaCI2 (**B**, PDB: 5IEC), and CirpT (**C**, PDB: 6RPT). Proteins are colored according to a gradient from *N*- to *C*-terminus.

Tick salivary lectin pathway inhibitors (TSLPIs) isolated from *Ixodes scapularis* and *Ixodes ricinus* (Schuijt et al., 2011) are part of a larger group of anticoagulant proteins. This group also includes Salp14, Salp9Pac, and Ixonnexin from *Ixodes scapularis* (Narasimhan et al., 2002; Assumpção et al., 2018), and BSAP1 and BSAP2 from *Ornithodoros savignyi* (Ehebauer et al., 2002). Investigation of BSAP1 by solution NMR spectroscopy shed light on the structure of these proteins (**Figure 4A**). BSAP1 embodies a rigid core consisting of ~60 amino acid residues stabilized by six conservative cysteines linked in three disulfide bonds (Denisov et al., 2019b). The core is composed of loops arranged in two layers: the first contains a major loop with an antiparallel β-strand, to which the *N*-terminus is connected by a disulfide bond, the second contains a minor loop and the *C*-terminal region stabilized by two disulfide bonds (Denisov et al., 2021). The overall fold can be distantly attributed to non-canonical EGF-like domain proteins. In contrast to the core, the *N*-terminal region of these proteins remains unstructured and shows high variation of amino acids composition, including basic (Salp14, Ixonnexin) and acidic tails (BSAP1) as well as the absence of an extended *N*-terminus (TSLPI, Salp9Pac). It has been shown that anticomplement activity is provided by the protein core, while anticoagulant activity is determined by the *N*-terminal region which is only present in the case of basic tail proteins such as Salp14 (Denisov et al., 2021). Inhibition of the lectin pathway is driven by blocking the binding of mannan-binding lectin (MBL) to mannan (Schuijt et al., 2011; Denisov et al., 2021). TSLPI has attracted particular attention as it has been shown to facilitate the transmission of *Borrelia* (Schuijt et al., 2011; Wagemakers et al., 2016) and therefore could be used as target for immunization.

A complement inhibitor with a unique structural fold has been identified in the *Rhipicephalus appendiculatus* transcriptome (Jore et al., 2016). *Rhipicephalus appendiculatus* complement inhibitor (RaCI)1 is an ~8 kDa protein that binds C5 protein blocking complement activation. From a structural point of view, RaCI1 and its homologue RaCI2 resemble snake toxins and consist of two loops connected to each other by three disulfide bonds (**Figure 4B**). Another complement inhibitor is CirpT, which stands for complement inhibitor from *Rhipicephalus pulchellus* of the terminal pathway (Reichhardt et al., 2019). CirpT adopts a 2-domain structure where the flat C-terminal domain is connected to the bulkier N-terminal “β-sandwich” domain by four disulfide bonds (**Figure 4C**). Although CirpT binds C5 in the same way as described above OmCI and RaCI1, all three proteins bind different C5 domains presenting distinct mechanisms of complement inhibition (**Figure 3B**).

Anticomplement proteins from *Ixodes scapularis* and *Ixodes ricinus* – ISACs, IRACs, IxACs, and Salp20 – belong to another class of tick salivary complement inhibitors which block the alternative pathway (Valenzuela et al., 2000; Daix et al., 2007; Tyson et al., 2007; Couvreur et al., 2008). Structural information about these proteins is very scarce and it is only known that the sequences contain four conservative cysteines (Daix et al., 2007). Although predicted molecular masses are ~20 kDa, native tick proteins are highly glycosylated with *N*- and *O*-linked glycans accounting for nearly half of the total molecular weight (Valenzuela et al., 2000; Tyson et al., 2007). Salp20 inhibits the alternative complement pathway by interacting with properdin and causing its dissociation from C3 convertase (Tyson et al., 2008). Salp20 administration has been shown to inhibit the alternative pathway-dependent pathogenesis in murine models of asthma and abdominal aortic aneurysm (Hourcade et al., 2016).

## Concluding Remarks

Over the past two decades, our knowledge of tick saliva composition has improved drastically, mainly due to numerous sialomes deposited in databanks (Martins et al., 2021). This has provided a wealth of material for sequence mining of polypeptides and proteins with a wide range of biological activities, including immunomodulation, for therapeutics and vaccine development. However, despite great expectations at the beginning of this century, we have to admit that the potential of tick as a source of bioactive compounds is far from being fully realized. Gavac^®^ (available in Latin American countries) and TickGARD^®^ (currently discontinued) are the only anti-tick vaccine on the market, which are based on the tick midgut protein BM86 (Schetters et al., 2016). Lipocalins nomacopan and сoversin are rare examples of the tick-derived therapeutics that reached phase III in clinical trials.

In our opinion, structural biology research can facilitate progress in the field. Detailed structural analysis could elucidate the molecular mechanism of proteins, provide insight into rational protein modification to adjust their properties, identify active epitopes for the development of synthetic vaccines, etc. Currently, structural information about tick proteins is very scarce. Indeed, only 30 unique structures of tick salivary proteins have been deposited in the RCSB database, 19 of which belong to well-known superfamilies: cystatin, serpin, lipocalin, and Kunitz-type domain. Taking into account that the recent analysis of sialomic data has revealed the presence of 45 thousands tick salivary proteins from 136 families (Ribeiro and Mans, 2020), we have barely scratched the surface of what ticks can offer us. The reason for this notorious scarcity of information is often the nature of proteins that present a real challenge to structure elucidation both by X-ray crystallography and NMR spectroscopy.

Due to secreted nature of salivary proteins, they often contain several disulfide bonds to ensure higher stability, and therefore obtaining proteins with the right disulfide connectivity is the first and possibly the most crucial step for not only structural but any other type of research. While that could be achieved using eukaryotic expression systems, tick proteins are often highly glycosylated (Tyson et al., 2007; Dias et al., 2009), which could hinder crystallization for X-ray crystallography and lead to spectral line broadening in NMR spectroscopy experiments. The correct disulfide connectivity could be achieved in *E.coli* using Origami strains, which greatly enhances disulfide bond formation due to mutated thioredoxin and glutathione reductases (Orrapin et al., 2019). In general, expressed proteins must be refolded in the presence of a redox couple, but often it requires tedious screening for optimal conditions and should be performed under the tight control of LC-MS analysis to obtain the homogeneous protein in the correct conformation. Folding can also be facilitated by substitution of one or several cysteines by selenocysteines, which increases the folding rate and leads to the native protein conformation (Steiner et al., 2012; Denisov et al., 2019b). As a last resort, proper disulfide bond formation could be achieved by regioselective oxidation of synthetic proteins as recently demonstrated on EVA-3 (Katayama and Nagata, 2021). Chemical synthesis could also be used to provide D-amino acids isomers of tick proteins for racemic X-ray crystallography, which greatly facilitate crystallization of proteins (Kent, 2018).

In conclusion, hundreds of millions of years of evolution have yielded nearly ideal proteins in tick saliva in terms of bioactivity, immunogenicity, and stability. Therefore, the elucidation of not only the biological function, but also their structures would allow us to understand their activity more completely, rationally change them and further use for our own purposes.

## Author Contributions

SD has gathered the literature. SD and ID have written and edited the manuscript. All authors contributed to the article and approved the submitted version.

## Funding

The Netherlands Organisation for Scientific Research and Maastricht UMC+ are greatly acknowledged for the financial support (NWO-ECHO 711.018.005 to ID and MUMC+ Kootstra Talent Fellowship to SD).

## Conflict of Interest

The authors declare that the research was conducted in the absence of any commercial or financial relationships that could be construed as a potential conflict of interest.

## Publisher’s Note

All claims expressed in this article are solely those of the authors and do not necessarily represent those of their affiliated organizations, or those of the publisher, the editors and the reviewers. Any product that may be evaluated in this article, or claim that may be made by its manufacturer, is not guaranteed or endorsed by the publisher.
